# Chiroptical property enhancement of chiral Eu(III) complex upon association with DNA-CTMA

**DOI:** 10.1038/s41598-020-75808-w

**Published:** 2020-11-03

**Authors:** Haruki Minami, Natsumi Itamoto, Wataru Watanabe, Ziying Li, Kazuki Nakamura, Norihisa Kobayashi

**Affiliations:** Graduate School of Science and Engineering, Chiba University, 1-33 Yayoi-cho, Inage-ku, Chiba, 263-8522 Japan

**Keywords:** DNA and RNA, Ligands, Photochemistry

## Abstract

DNA-based materials have attracted much attention due to their unique photo-functional properties and potential applications in various fields such as luminescent and biological systems, nanodevices, etc. In this study, the photophysical properties of a chiral Eu(III) complex, namely (Eu(*D*-facam)_3_), within DNA films were extensively investigated. The enhancement of photoluminescence (more than 25-folds increase of luminescence quantum yield) and degree of circularly polarization in luminescence (*g*_lum_ = − 0.6) was observed upon interaction with DNA. Various photophysical analyses suggested that the emission enhancement was mainly due to an increase of the sensitization efficiency (high *η*_sens_) from the ligands to Eu(III) and suppression of the vibrational deactivation upon immobilization onto the DNA molecule. From CD and VCD measurements, it was suggested that the coordination structure of Eu(*D*-facam)_3_ was affected by the interaction with DNA, suggesting that the structural change of Eu(*D*-facam)_3_ contributed to the improvement of its luminescent properties.

## Introduction

In recent years, biopolymer-based materials have attracted much attention for their unique properties and potential applications as photo-functional materials due to their highly ordered structures^1–3^. In particular, DNA possesses the unique ability to incorporate various types of functional materials like metal complexes^4,5^, organic dyes^6,7^, and conductive polymers^8^, thus leading to an enhancement of their photo-functional properties. This ability can be mainly attributed to the electrostatic properties of the phosphate group, selective affinity for small molecules by intercalation and binding of specific molecules into its grooves^9–11^. A solid matrix made of DNA and cetyltrimethylammonium chloride (CTMA) was widely investigated as a polar, organic solvent soluble complex since natural DNA can be solubilized only in water^12–15^. To date, various optoelectronic devices that utilize this DNA-surfactant complex such as optical amplifiers^16–19^, organic light emitting diodes (OLEDs)^20^, photodetectors^21^ and organic transistors^22^ have been reported. We also described DNA-based transistor memories, color tunable OLEDs and electrochemiluminescent devices exploiting the unique features of DNA-based functional materials^23–27^.

Aiming to develop improved DNA-based photo-functional materials, this study focuses on DNA/Eu(III) complexes in view of their characteristic optical properties. In fact, Eu(III) complexes are promising candidates for such purpose since they are strong luminophores with high color purities and long emission lifetimes^28^. In addition, chiral Eu(III) complexes exhibit chiral optical properties like circular dichroism (CD) and circularly polarized luminescence (CPL)^29,30^. CPL, which corresponds to the luminescence generated in response to electromagnetic waves with different rotation, provides advanced information based on the rotation of light. The CPL is expected to not only improve the precise sensing of chiral molecules and biomolecules as well as structural analyses of biopolymers but also lead to the development of multifunctional displays, security paints and optical communication^31–33^. Currently, CPL is obtained by using optical devices such as a combination of linear polarizer and quarter wave retarder^34^. However, the reduction in emission intensity remains an intrinsic shortcoming. Luminescent materials that do not require additional optical apparatuses to generate CPL are therefore in demand. Typically, chiral organic luminophores and transition metal complexes display a strong luminescence; however, the degree of polarization of the luminescence is considerably lower than that of chiral Eu(III) complexes. The parameter *g*_lum_ is generally used as a dissymmetry ratio of the emission and is defined as *g*_lum_ = 2(*I*_L_ − *I*_R_)/(*I*_L_ + *I*_R_), where *I*_L_ (*I*_R_) is the intensity of left (right) circularly polarized luminescence. Theoretically, *g*_lum_ can be defined as *g*_lum_ = 4 (|*m*|/|*μ*|)·cos τ, where *m* and *μ* are the magnetic and electric dipole transition moments, respectively and τ is the angle between them^35^. For organic luminophores, a large |*μ*| owing to the allowed *π*–π^*^ transition may lead to a high luminescence quantum yield, while maintaining a low *g*_lum_. In contrast, the luminescence deriving from Eu(III) complexes can be attributed to the forbidden f–f transitions and their low |*μ*| results in a high *g*_lum_. Therefore, the simultaneous achievement of a strong emission intensity and high *g*_lum_ seems challenging. On the other hand, the emission enhancement of luminophores due to their association with DNA was widely reported^36–39^. In our previous study, emission enhancement and induced CPL were achieved by associating an achiral Eu(III) complex with DNA-CTMA^40^. Therefore, a more distinctive enhancement of the optical properties can be expected by adding chiral sites to the Eu(III) complex, which interacts with DNA.

In this study, we investigated the luminescence properties of a chiral Eu(III) complex within a DNA film. To this aim, we selected Eu(*D*-facam)_3_ (europium tris[3-(trifluoromethylhydroxymethylene)-(+)-camphorate]) as chiral Eu(III) complex, which is known for its use as NMR-shift reagent and biological sensing probe^41,42^. Interestingly, a higher luminescence intensity and |*g*_lum_| of CPL were achieved from Eu(*D*-facam)_3_ compared with the conventional polymer upon interaction with DNA.

## Results and discussion

### Interaction between the chiral Eu(III) complex and DNA-CTMA

**Figure 1:**
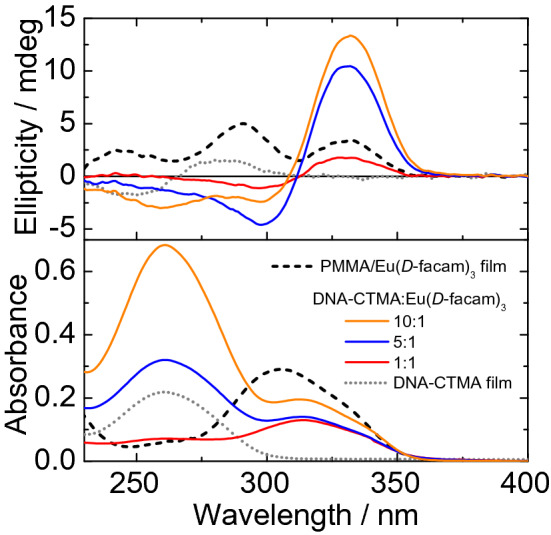
Absorption (bottom) and circular dichroism (CD, top) spectra of DNA-CTMA/Eu(*D*-facam)_3_ films at various Eu(*D*-facam)_3_:DNA-CTMA molar ratios and PMMA/Eu(*D*-facam)_3_ film.

First, we introduced Eu(*D*-facam)_3_ into DNA backbone to observe their optical properties. Since DNA is soluble only in water whereas Eu(*D*-facam)_3_ is insoluble in water, DNA is modified with CTMA, which is one of the most typical surfactants utilized for biomolecules. By utilizing DNA-CTMA, we successfully fabricated DNA-CTMA/Eu(*D*-facam)_3_ films. The absorption and CD spectra of the DNA-CTMA/Eu(*D*-facam)_3_ films at various Eu(*D*-facam)_3_:DNA-CTMA molar ratios are shown in Fig. 1. For comparison, the optical analysis of a PMMA/Eu(*D*-facam)_3_ film was also conducted; PMMA has no chirality in its structure, while DNA has a well-known axisymmetric helical structure^43^. In the PMMA/Eu(*D*-facam)_3_ film, an absorption band assignable to the π–π* transition of the β-diketonate^44^ in the *D*-facam was observed around 305 nm. On the other hand, the absorption peaks of the DNA-CTMA/Eu(*D*-facam)_3_ films were red-shifted by about 10 nm compared to that of the PMMA/Eu(*D*-facam)_3_ film, suggesting an interaction between DNA and Eu(*D*-facam)_3_. It is possible that Eu(*D*-facam)_3_ electrostatically approach the anionic phosphate groups in the DNA backbone, and such absorption change suggests the intercalation or semi-intercalation between base pairs subsequent by electrostatic interaction^45,46^. The CD spectrum indicates ellipticity at each wavelength^29^. Ellipticity is proportional to the difference of absorbance against the left-handed and right-handed circularly polarized light, thus a change in ellipticity indicates the structural chirality of the molecules corresponding to its absorption. In the PMMA/Eu(*D*-facam)_3_ film, a positive Cotton effect corresponding to the absorption band of *D*-facam was observed. This was attributed to the fact that the *D*-facam ligand of Eu(*D*-facam)_3_ possesses a chiral structure. For the DNA-CTMA/Eu(*D*-facam)_3_ films, typical exciton-splitting CD signals with positive (330 nm) and negative (300 nm) Cotton effects centered at the absorption peak of the ligand were observed. This indicated that the exciton coupling of the *D*-facam ligands in Eu(*D*-facam)_3_ occurred upon interaction with DNA. In addition, the signal intensity of the exciton coupling increased relative to the increase of the DNA ratio, strongly indicating that the structural chirality of Eu(*D*-facam)_3_ was enhanced.

**Figure 2:**
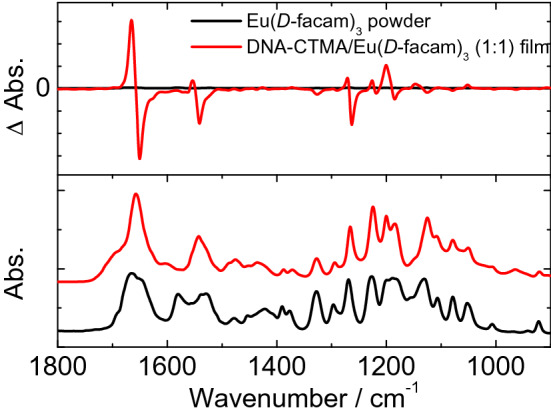
Infrared absorption (bottom) and vibrational circular dichroism (VCD, top) spectra of the Eu(*D*-facam)_3_ powder and DNA-CTMA/Eu(*D*-facam)_3_ film [Eu(*D*-facam)_3_:DNA-CTMA = 1:1].

Vibrational circular dichroism (VCD) spectroscopy experiments were carried out to determine more detailed structural change of Eu(*D*-facam)_3_ in the presence of DNA. Figure 2 shows the infrared absorption (IR) and VCD spectra of the Eu(*D*-facam)_3_ powder and DNA-CTMA/Eu(*D*-facam)_3_ film [Eu(*D*-facam)_3_:DNA-CTMA molar ratio was 1:1]. In the IR spectra (bottom), the absorption peaks corresponding to the C=O stretching vibration (around 1650 cm^−1^) and C=C stretching vibration (around 1500–1600 cm^−1^) of the *D*-facam ligand were observed for both the Eu(*D*-facam)_3_ powder and DNA-CTMA/Eu(*D*-facam)_3_ film^47^. In the VCD spectra (top), although no significant signal was observed in the case of the Eu(*D*-facam)_3_ powder, the VCD signals corresponding to the absorption bands of *D*-facam were observed for the DNA-CTMA/Eu(*D*-facam)_3_ film. Especially, the absorption band assignable to the stretching vibration of the C=O group that was in vicinity to the central Eu(III) ion showed a significant exciton splitting pattern, indicating that the coordination symmetry of the ligand field of Eu(III) might be affected. In view of the results of the CD and VCD measurements, it was suggested that the structure of Eu(*D*-facam)_3_ was distorted by the interaction with DNA, thus potentially affecting the luminescent properties of Eu(*D*-facam)_3_.

### Emission properties of the DNA-CTMA/Eu(***D***-facam)_3_ film

**Figure 3:**
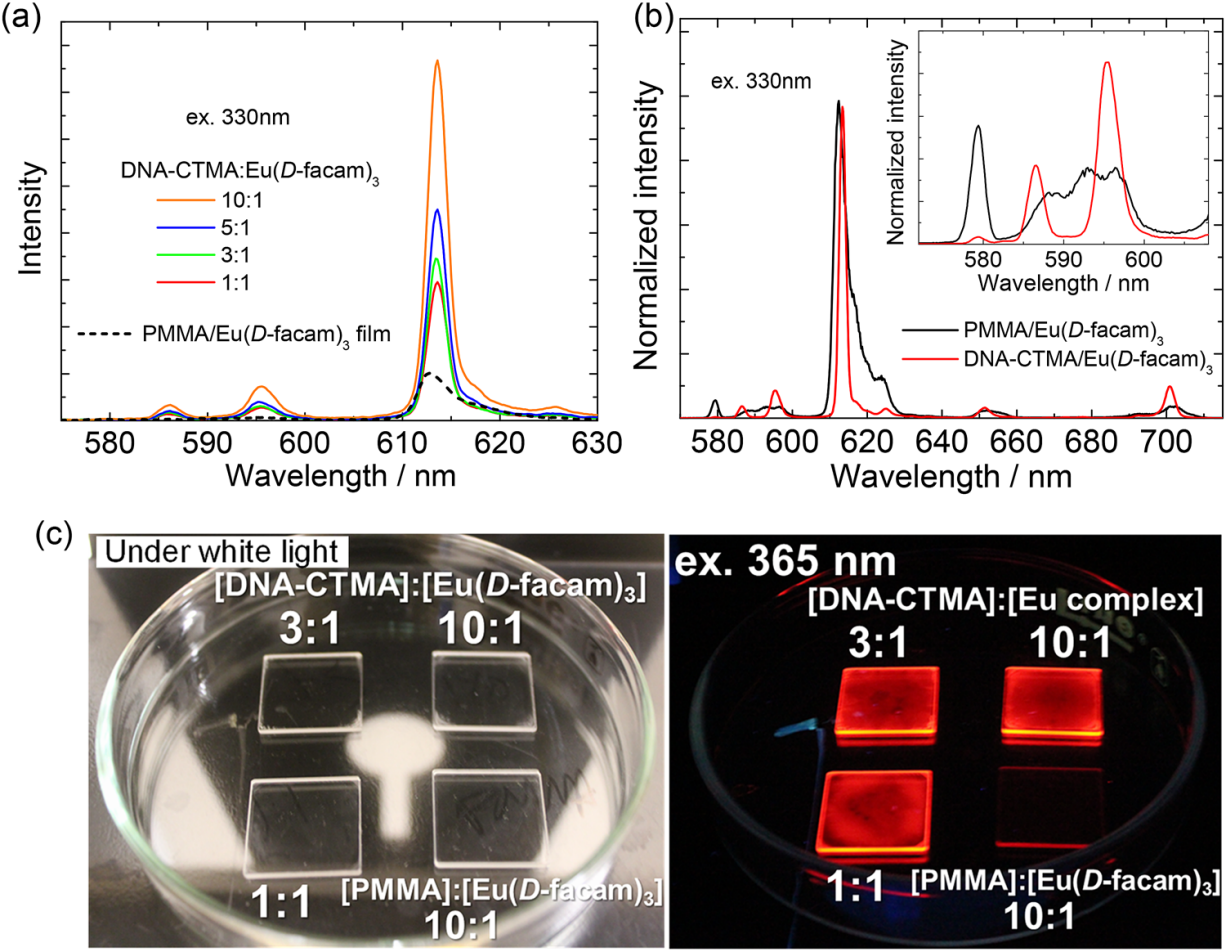
Emission spectra of DNA-CTMA/Eu(*D*-facam)_3_ films at various Eu(*D*-facam)_3_:DNA-CTMA molar ratios and PMMA/Eu(*D*-facam)_3_ film upon excitation at 330 nm, as shown by the original emission intensities (**a**) and normalized intensities (**b**). Photograph of the DNA-CTMA/Eu(*D*-facam)_3_ films and PMMA/Eu(*D*-facam)_3_ film (**c**).

In order to discuss the influence of the interaction between Eu(*D*-facam)_3_ and DNA-CTMA on the luminescent properties, the emission spectra of the PMMA/Eu(*D*-facam)_3_ and DNA-CTMA/Eu(*D*-facam)_3_ films were examined (Fig. 3). For all films, a red emission with sharp peaks due to the f–f transition of the Eu(III) ion was observed upon ligand excitation (330 nm). In the case of the PMMA/Eu(*D*-facam)_3_ film, emission peaks were observed at 579, 585–600 and 613 nm; they are assignable to the ^5^D_0_ → ^7^F_0_, ^5^D_0_ → ^7^F_1_ and ^5^D_0_ → ^7^F_2_ transition of Eu(III) ions, respectively^48^. Interestingly, for the DNA-CTMA/Eu(*D*-facam)_3_ film, the emission peak assignable to the ^5^D_0_ → ^7^F_1_ transition split into two peaks (586 and 595 nm). This change of the emission peak obviously indicated that the interaction with DNA affected the crystal field around the Eu(III) ion (Fig. 3b inset). In comparison to the PMMA/Eu(*D*-facam)_3_ film, the DNA-CTMA/Eu(*D*-facam)_3_ films showed a stronger emission. Their emission intensity was enhanced with increasing ratios of DNA-CTMA, suggesting that a non-radiative deactivation caused by a molecular vibration was suppressed due to the immobilization onto the DNA backbone^49,50^.

In regard to luminescence intensity of each emission band, radiative rate of the emission band is mainly determined by electric dipole transition which is sensitive to the ligand field (i.e. electric field around Eu(III) ion). Because the emission peaks assignable to the ^5^D_0_ → ^7^F_1_ transition derives from mainly magnetic dipole (MD) transition, its radiative rate is not considerably affected by the ligand field. Therefore, the symmetry of Eu(*D*-facam)_3_ can be discussed based on the ratio of the emission intensities obtained from the MD moment (*I*_MD_) and ED moment (*I*_ED_)^51^. The emission ratio (*I*_rel_ = *I*_ED_/*I*_MD_) of the PMMA/Eu(*D*-facam)_3_ and DNA-CTMA/Eu(*D*-facam)_3_ films are shown in Table 1. *I*_MD_ and *I*_ED_ were calculated by integrating the emission intensities at 582–600 nm and 605–630 nm, respectively. Since the values of *I*_rel_ for the DNA-CTMA/Eu(*D*-facam)_3_ films (5.14–5.60) were lower than that of the PMMA/Eu(*D*-facam)_3_ film (7.64), it was estimated that the coordination structure around the Eu(III) ion in Eu(*D*-facam)_3_ changed to a higher symmetric structure in the presence of DNA. The changes in the structure of Eu(*D*-facam)_3_ were also confirmed by CD and VCD measurements. These results clearly supported that Eu(*D*-facam)_3_ and DNA interacted with each other and led to a stronger emission, as shown in Fig. 3.

**Table 1:** Ratio of the emission intensity of the MD and ED moment (*I*_rel_), luminescence lifetime (*τ*), radiative rate (*k*_r_), non-radiative rate (*k*_nr_), intrinsic quantum yield of Eu(III) ion (*Φ*_Ln_), total quantum yield (*Φ*_tot_), and efficiency of sensitization (*η*_sens_) of the DNA-CTMA/Eu(*D*-facam)_3_ and PMMA/Eu(*D*-facam)_3_ films.

Sample	Polymer:Eu(III) (weight ratio of Eu(III))	*I*_rel_	τ (µs)	*k*_r_ (s^−1^)	*k*_nr_ (s^−1^)	$\it \Phi_{{{\text{Ln}}}}$ (%)	$\it \Phi_{{{\text{tot}}}}$ (%)	*η*_sens_ (%)
PMMA/Eu(III) film	− (12 wt%)	7.64	114	419	8353	4.77	0.5	10.5
DNA/Eu(III) film	1:1 (58 wt%)	5.15	626	317	1597	16.5	11.9	72.1
	3:1 (31 wt%)	5.14	606	316	1334	19.2	11.7	61.0
	5:1 (22 wt%)	5.62	601	341	1323	20.5	12.0	58.6
	10:1 (12 wt%)	5.60	602	340	1322	20.4	13.7	67.0

### Detailed analyses of photophysical parameters of the DNA-CTMA/Eu(***D***-facam)_3_ films

Generally, Eu(III) ions with a highly symmetrical structure hardly show any strong emission since the high symmetry of the Eu(III) ion results in a low radiative rate^52^. Therefore, we determined the factors that contributed to the emission enhancement of Eu(*D*-facam)_3_ in DNA-CTMA films. The total quantum yield (*Φ*_tot_) and luminescence lifetime (*τ*) of the PMMA/Eu(*D*-facam)_3_ and DNA-CTMA/Eu(*D*-facam)_3_ films were obtained (Table 1). The luminescent quantum yield was 0.5 and 11.9–13.7% for the PMMA/Eu(*D*-facam)_3_ and DNA-CTMA/Eu(*D*-facam)_3_ films, respectively; the luminescent quantum yield of Eu(*D*-facam)_3_ was significantly increased in the case of DNA-CTMA compared to PMMA. The radiative rate constants of Eu(III) ions (*k*_r_) was also calculated using the relation:

$$k_{{\text{r}}} = A_{{{\text{MD}},0}} \times n^{{3}} \times \left( {\frac{{I_{{{\text{tot}}}} }}{{I_{{{\text{MD}}}} }}} \right)$$

where *A*_MD,0_ is the spontaneous emission coefficient of the ^5^D_0_ → ^7^F_1_ transition (= 14.65 s^−1^), *n* is the refractive index of the medium and *I*_tot_/*I*_MD_ is the ratio of the integrated radiation corresponding to the ^5^D_0_ → ^7^F_j_ transition (j = 0–6) to the peak area corresponding to the ^5^D_0_ → ^7^F_1_ transition^51,53^. Here, the value of* n* was determined to be 1.52 and 1.49 for the DNA-CTMA^54^ and PMMA solid film^55^, respectively. The non-radiative rate constant (*k*_nr_) can be calculated from the relations:

$$\tau = \frac{1}{{k_{{\text{r}}} + k_{{{\text{nr}}}} }},\quad k_{{{\text{nr}}}} = \frac{1}{\tau } - k_{{\text{r}}}$$

while the intrinsic quantum yield (*Φ*_Ln_) and efficiency of sensitization of the lanthanide luminescence by the ligand (*η*_sens_) can be calculated from the relations^56^:

$$\Phi_{{{\text{Ln}}}} = \frac{{k_{{\text{r}}} }}{{k_{{\text{r}}} + k_{{{\text{nr}}}} }},\quad \eta_{{{\text{sens}}}} = { }\frac{{\Phi_{{{\text{tot}}}} }}{{\Phi_{{{\text{Ln}}}} }}$$

These photophysical parameters are listed in Table 1. It was evident that the *k*_r_ value of Eu(*D*-facam)_3_ for the DNA-CTMA films (316–340 s^−1^) was lower than that of the PMMA film (419 s^−1^). This implies that the probability of light emission from the excited state decreased in the presence of DNA, which is consistent with the structural change around the Eu(III) ion towards a higher symmetry structure, as discussed above. On the other hand, *k*_nr_ decreased and *η*_sens_ increased for the DNA-CTMA films compared to the PMMA film. The *k*_nr_ value of PMMA was approximately 8400 s^−1^; it decreased to 1300 s^−1^ upon mixing with DNA. It is known that the molecular vibration can be suppressed when the molecules are immobilized on a DNA structure^49,50^. Therefore, the decrease in *k*_nr_ clearly indicated a suppressed vibrational deactivation of the excited states of the Eu(III) ion due to the immobilization on a DNA molecule. The decreased *k*_nr_ contributed to improving the intrinsic quantum yield (*Φ*_Ln_); the calculated *Φ*_Ln_ increased from 4.77% (in PMMA) to 20.5% (in DNA-CTMA). In addition, the *η*_sens_ value of Eu(*D*-facam)_3_ was significantly improved upon interaction with DNA; it was almost 6 times higher compared to that of PMMA. This improvement of *η*_sens_ is believed to contribute to the emission enhancement of Eu(*D*-facam)_3_.

**Figure 4:**
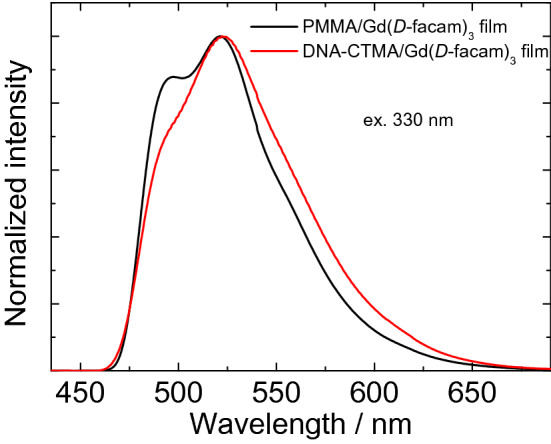
Phosphorescence spectra of the DNA-CTMA/Gd(*D*-facam)_3_ and PMMA/Gd(*D*-facam)_3_ films at 77 K.

It is known that the *η*_sens_ value of lanthanide complexes significantly depends on the relationship between the T_1_ level of the ligands and the accepting 4f level of the central metal ion^56^. An adequate energy gap between the T_1_ and 4f levels facilitates the energy transfer from the ligands to the metal ion. However, a close match between the T_1_ and 4f levels should be avoided as it induces back energy transfer from the metal ion to the ligands. To investigate the change of the T_1_ level of the *D*-facam ligand in the different polymers, the phosphorescence of Gd(*D*-facam)_3_ of the PMMA and DNA-CTMA film was measured at 77 K (Fig. 4)^57^. It is known that the first excitation energy of Gd(III) is much higher than the triplet energy of general complex ligands^58^. Thus, the energy transfer from ligands to the Gd(III) ion hardly occur. Only the phosphorescence from the ligands should be observed. The T_1_ level can be calculated from the onset wavelength of the phosphorescence spectrum. Broad phosphorescence bands of *D*-facam were observed around 460–650 nm for both films. The onset wavelength of the phosphorescence spectrum of each film almost matched (462 nm), indicating that the T_1_ level of *D*-facam was unperturbed (calculated as ca. 21,600 cm^−1^). Thus, the improvement of *η*_sens_ was caused by factors other than the change in the T_1_ level of the ligands. Therefore, the changes of the distance between Eu(III) and ligands as well as the angle between them, as evidenced by the CD and VCD spectra and *I*_rel_ values, contributed to the significant improvement of *η*_sens_. In addition, the decrease of *k*_nr_ discussed above obviously indicates that Eu(*D*-facam)_3_ was tightly immobilized onto the DNA structure. The immobilization of these molecules might suppress the vibrational deactivation of the T_1_ states, and allow to improve *η*_sens_, which represents the energy transfer efficiency from the ligands to the central metal ion.

### Circularly polarized luminescence induced by DNA-CTMA

**Figure 5:**
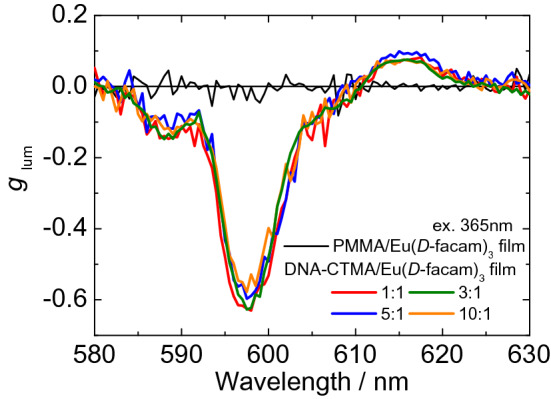
CPL spectra of the DNA-CTMA/Eu(*D*-facam)_3_ films at various Eu(*D*-facam)_3_:DNA-CTMA molar ratios and PMMA/Eu(*D*-facam)_3_ film upon excitation at 330 nm.

As discussed above, it was demonstrated that the structural chirality of Eu(*D*-facam)_3_ significantly changed upon its interaction with DNA-CTMA. Therefore, it was assumed that CPL, which reflects the chiral luminescence, might also be greatly enhanced^59^. Therefore, we measured the CPL spectra of the PMMA/Eu(*D*-facam)_3_ and DNA-CTMA/Eu(*D*-facam)_3_ films and their *g*_lum_ values are shown in Fig. 5. An emission dissymmetry factor [*g*_lum_ = 2(*I*_L _− *I*_R_)/(*I*_L_ + *I*_R_)] was utilized to quantitatively evaluate the magnitude of CPL, where *I*_L_ and *I*_R_ represent the emission intensity of left-handed and right-handed circular polarized luminescence, respectively^35^. For the PMMA/Eu(*D*-facam)_3_ film, the CPL intensity was very weak, and the *g*_lum_ was calculated to be − 0.02 at ^5^D_0_ → ^7^F_1_ (598 nm, MD transition). On the other hand, in the case of the DNA-CTMA/Eu(*D*-facam)_3_ films, clear CPL signals were observed at ^5^D_0_ → ^7^F_1_ (598 nm, MD transition) and ^5^D_0_ → ^7^F_2_ (615 nm, ED transition). The *g*_lum_ at ^5^D_0_ → ^7^F_1_ (598 nm) was determined to be − 0.62, which resulted approximately 30 times enhanced compared to the PMMA/Eu(*D*-facam)_3_ film. Such chirality enhancement of the luminescence was supposed to be due to the change of the ligand field of Eu(*D*-facam)_3_ caused by the interaction with DNA-CTMA.

## Conclusion

In summary, the change of the photophysical properties of Eu(*D*-facam)_3_ in the DNA films was investigated in detail. An emission enhancement and higher dissymmetry factor (− 0.6) were observed upon interaction with DNA. Various photophysical analyses suggested that the emission enhancement was mainly due to the increase of the sensitization efficiency (high *η*_sens_) from the ligands to Eu(III) as well as suppression of the vibrational deactivation upon immobilization onto the DNA molecule. These phenomena were primarily driven by the transformation of the coordination structure of Eu(*D*-facam)_3_ upon association with DNA. It can be assumed that such enhancement of the optical properties of Eu(III) complexes with DNA can contribute to the development of not only luminescent devices, nanodevices and catalysts but also applications related to biological fields and DNA engineering.

## Methods

### Reagents

All chemicals were commercially available and used as received. Europium tris[3-(trifluoromethylhydroxymethylene)-(+)-camphorate] (Eu(*D*-facam)_3_), (+)-3-(trifluoroacetyl)camphor, and poly(methylmethacrylate) (PMMA, Mw: ~ 350,000) were purchased from Sigma-Aldrich (USA). Gadolinium(III) acetate hydrate was purchased from FUJIFILM Wako Pure Chemical Corporation (Japan). The sodium salts of DNA (base pairs: ca. 10,000) were provided by Nippon Chemical Feed Co., Ltd. (Japan). These were marine-based salts that were first isolated from frozen salmon milt through a homogenization process followed by removal of proteins and impurities. Cetyltrimethylammonium chloride (CTMA, 98% purity) and 1-butanol were purchased from Tokyo Chemical Industry Co., Ltd (Japan).

### Preparation of the DNA-CTMA complex

DNA-CTMA was prepared by precipitating DNA with a cationic surfactant complex of CTMA in water through an ion exchange reaction that replaced the sodium cations of the DNA. The DNA complex with CTMA (DNA-CTMA) was prepared by the addition of a 10 mM aqueous solution of DNA (based on the concentration of the phosphate groups) to a 10 mM CTMA solution. The precipitate was filtered and thoroughly washed with ultrapure water and then dried in vacuo. The resulting DNA-CTMA was more water insoluble and more mechanically stable than the DNA itself due to the long alkyl chain of the CTMA. Through the formation of the CTMA complex, DNA-CTMA was soluble in solvents more compatible with device fabrication, such as chloroform, ethanol, methanol, butanol, or a chloroform/alcohol blend.

### Preparation of DNA-CTMA/Eu(***D***-facam)_3_ films

DNA-CTMA/Eu(*D*-facam)_3_ solutions were prepared by dissolving DNA-CTMA and Eu(*D*-facam)_3_ in 1-butanol. The concentration of DNA-CTMA and Eu(*D*-facam)_3_ were set to 0.1–1.0 mmol/L and 0.1 mmol/L, respectively. The concentration ratio of DNA-CTMA to Eu(*D*-facam)_3_ was varied by changing the DNA-CTMA concentration (based on the concentration of the phosphate groups) in solution. The DNA-CTMA/Eu(*D*-facam)_3_ films were prepared by casting 200 μL of these solutions onto quartz substrates (2 × 2 cm^2^). The weight percentages of Eu(*D*-facam)_3_ in the DNA-CTMA films ranged between 12 and 58 wt%. A PMMA film containing Eu(*D*-facam)_3_ (12 wt%) was also prepared for comparison.

### Measurements of the optical properties

The absorbance and CD spectra of the DNA-CTMA/Eu(*D*-facam)_3_ and PMMA/Eu(*D*-facam)_3_ films were acquired using a photonic multichannel analyzer (J-1100, JASCO Corporation, Japan). The emission spectra were acquired using a spectrofluorometer (FP-6600, JASCO Corporation, Japan). The emission quantum yields were calculated from the data obtained from an absolute PL quantum yield spectrometer (Quantaurus-QY C11347-01, Hamamatsu photonics K. K., Japan). The emission lifetimes were determined using a time-resolved fluorescence spectrometer (Quantaurus-Tau C11367-21, Hamamatsu photonics K. K., Japan). CPL measurements were conducted using a previously reported system^40,60^, which consisted of the following components: 375 nm LED (M365L2, Thorlabs Japan Inc., Japan), LED driver (DC2100, Thorlabs Japan Inc., Japan), photoelastic modulator (PEM-90, Hinds instruments, Inc. United States), photomultiplier tube (H7732-10, Hamamatsu photonics K. K., Japan), linearly polarized cubic prism (200,000:1), photomultiplier tube (H7732-10, Hamamatsu photonics K. K., Japan), and dual phase DSP lock-in amplifier (7265, Signal Recovery Ltd., United Kingdom). The appropriate detection wavelength of the monochromator and PEM was controlled by a PC.
